# Dietary factors and MRI-Defined synovial inflammation progression in knee osteoarthritis: a longitudinal mixed-effects analysis from the osteoarthritis initiative

**DOI:** 10.3389/fragi.2026.1877396

**Published:** 2026-07-29

**Authors:** Yin Wang, Ming Chen

**Affiliations:** 1 Trauma Orthopedics Center, Wuhan Fourth Hospital, Wuhan, Hubei, China; 2 Hubei Provincial Clinical Research Center for Orthopaedics, Wuhan, Hubei, China

**Keywords:** diet, effusion-synovitis, knee osteoarthritis, longitudinal study, mixed-effects model, MRI, osteoarthritis initiative, synovial inflammation

## Abstract

**Background:**

Dietary factors have been implicated in the development and progression of knee osteoarthritis (KOA), yet their role in modulating synovial inflammation remains unclear, particularly when assessed using quantitative MRI. This study aimed to investigate the cross-sectional and longitudinal associations between dietary intake and MRI-defined effusion-synovitis (ES) in knees at risk of KOA.

**Methods:**

This was a secondary analysis based on the Pivotal OAI MR Imaging Analyses incidence OA (POMA_INCOA) sub-cohort. Dietary exposures were derived from the Block Brief 2000 food frequency questionnaire. The primary outcomes were baseline ES volume and 12-month change in ES volume (ΔES). Linear mixed-effects models with participant-specific random intercepts were used to account for within-subject correlation due to bilateral knees. Models were adjusted for age, sex, body mass index, baseline ES volume (for longitudinal models), and Kellgren-Lawrence grade (KLG). Sensitivity analyses included additional adjustment for physical activity and diabetes, as well as alternative outcome specifications (winsorization and categorical progression models).

**Results:**

A total of 659 knees from 620 participants were analyzed. In cross-sectional analyses, a higher intake of meat, fish, poultry, eggs, and legumes was significantly associated with a larger baseline ES volume (β = 0.452, 95% CI: 0.075 to 0.829, p = 0.019), though this association did not retain statistical significance after stringent FDR correction (FDR-adjusted p > 0.050). In longitudinal analyses, no dietary variables, including percentage of energy from fat (β = −0.005, 95% CI: −0.150 to 0.006, p = 0.383), demonstrated a statistically significant association with 12-month ΔES. This lack of longitudinal association remained consistent across all sensitivity analyses, including models adjusted for physical activity and diabetes, and those using winsorized or binary progression outcomes (all p > 0.05).

**Conclusion:**

While specific protein-rich food groups are cross-sectionally linked to baseline synovial inflammation volume, dietary factors are not robustly associated with the short-term progression of effusion-synovitis in knees at risk of KOA. These findings suggest that dietary intake may correlate with the inflammatory “set-point” of the joint but has limited influence on active 12-month inflammatory fluctuations, highlighting the complexity of inflammatory mechanisms in early KOA.

## Introduction

Dietary factors have been extensively investigated in relation to knee osteoarthritis (KOA), with accumulating evidence suggesting that dietary patterns and nutrient intake may influence disease risk, symptoms, and structural progression through metabolic and inflammatory pathways (Sophocleous, 2023; Wei and Dai, 2022). Diets high in saturated fat and refined carbohydrates have been associated with systemic low-grade inflammation (Shawl et al., 2024; Kasprzyk et al., 2025), whereas diets rich in fruits, vegetables, and antioxidants are thought to exert anti-inflammatory effects. However, most previous studies have focused on clinical outcomes, such as pain or incident radiographic KOA, rather than imaging-based inflammatory phenotypes (Guermazi et al., 2013). Given that KOA is highly prevalent in aging populations and represents a major contributor to age-related disability (Gelber, 2024; Kloppenburg et al., 2025), a better understanding of the relationship between diet and early joint inflammation is of substantial clinical and public health importance.

Importantly, the relationship between dietary intake and synovial inflammation, particularly as quantified by Magnetic Resonance Imaging (MRI), remains poorly understood (Bruder et al., 2026). It is unclear whether dietary factors can meaningfully influence short-term changes in effusion-synovitis, especially in individuals at risk of KOA prior to established structural disease. Addressing this gap is critical for determining whether dietary modification could serve as a viable strategy for targeting early inflammatory processes in KOA.

The Osteoarthritis Initiative (OAI) (Schneider et al., 2008) provides a unique opportunity to investigate these questions through its well-characterized longitudinal cohort with standardized MRI assessments and detailed dietary data. The Pivotal OAI MR Imaging Analyses (POMA) incidence OA sub-cohort further enables evaluation of early disease processes in knees at risk of developing KOA, offering a valuable framework for studying inflammation preceding structural progression (Roemer et al., 2015).

Therefore, the aim of this study was to examine the longitudinal associations between dietary factors and MRI-defined effusion-synovitis progression over 12 months in knees at risk of KOA. Using a knee-level analytical framework and linear mixed-effects models (LMMs) (Carnoli et al., 2024) to account for within-subject correlation, we sought to determine whether dietary intake is independently associated with short-term changes in synovial inflammation.

## Methods

### Study design and population

This study was a longitudinal, knee-level secondary analysis based on data from the Pivotal OAI MR Imaging Analyses incidence OA (POMA_INCOA) sub-cohort of the OAI, a multicenter, prospective study designed to investigate the development and progression of KOA (ClinicalTrials.gov identifier: NCT00080171). The OAI incidence sub-cohort includes individuals without symptomatic KOA at baseline but at increased risk of developing the disease.

The POMA_INCOA sub-cohort represents an imaging-enriched sample of knees without radiographic KOA at baseline, including knees that developed incident KOA during follow-up and matched knees that did not. Although this sub-cohort was originally constructed using a nested case-control design, the present analysis did not preserve the original sampling framework. Instead, all eligible knees with complete baseline dietary data, MRI measurements, and covariates were included and analyzed as a longitudinal observational sample.

**FIGURE 1:**
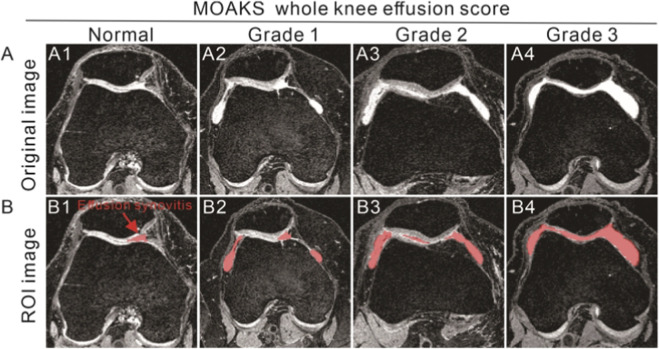
MRI-based segmentation of effusion-synovitis across different MOAKS grades. **(A)** Representative sagittal knee MRI images corresponding to MOAKS effusion-synovitis grades 0 to 3 **(A1–A4)**. **(B)** Corresponding region-of-interest (ROI) images illustrating semi-automated segmentation of effusion-synovitis for each grade **(B1–B4)**. All images were acquired using a sagittal three-dimensional dual-echo in steady-state (DESS) sequence with water excitation. The semi-quantitative MOAKS tiers (Grades 0–3) serve strictly as descriptive visual anchors to demonstrate the segmentation pipeline’s coverage across a spectrum of inflammatory severities. The segmented regions represent areas of effusion-synovitis used for quantitative volume assessment. Abbreviations: MOAKS, MRI Osteoarthritis Knee Score; MRI, magnetic resonance imaging; ROI, region of interest.

The unit of analysis was the knee; therefore, participants could contribute one or both knees. Knees with available baseline and 12-month MRI assessments of effusion-synovitis volume were included (Figure 1). To ensure that inflammatory changes preceded structural progression, knees that developed incident KOA within the first 12 months were excluded. The study flowchart is presented in Supplementary Figure S1.

### Outcome assessment

The primary outcome was the 12-month change in effusion-synovitis volume (ΔES), defined as:

$$\Delta ES={ES}_{12month}-{ES}_{baseline}$$

Effusion-synovitis volume was quantified using semi-automated segmentation of fluid-equivalent signal intensity on a sagittal three-dimensional dual-echo in steady-state (DESS) MRI sequences, implemented in OsiriX software, as previously described (Wang et al., 2016a). The imaging readers were entirely blinded to all participant baseline clinical data, KLG, and dietary patterns. To minimize measurement error and avoid partial volume blending artifacts common at tissue interfaces, all initially generated high-signal masks were subsequently reviewed and manually refined on a slice-by-slice basis by a senior orthopedic specialist (M.C., with over 10 years of extensive experience in musculoskeletal MRI interpretation). This expert tracing guaranteed a clear anatomical demarcation, manually excluding any overlapping high-signal pixels belonging to the adjacent articular cartilage plates or the joint capsule, thereby ensuring high reproducibility of the volumetric assessment. The intra-observer intraclass correlation coefficient (ICC) for the primary senior reader (M.C.) was 0.965 (95% CI: 0.941–0.978), and the inter-observer ICC between two independent readers was 0.938 (95% CI: 0.902–0.959), indicating excellent volumetric reproducibility. This approach allows for quantitative assessment of synovial inflammation and has been validated for evaluating longitudinal changes in effusion-synovitis.

In sensitivity analyses, ΔES was additionally (1) winsorized at the 1st and 99th percentiles to reduce the influence of extreme values and (2) dichotomized to define effusion-synovitis progression (ΔES >0 vs. ≤ 0).

### Dietary assessment

Baseline dietary intake was assessed using the Block Brief 2000 food frequency questionnaire (FFQ), which captures habitual dietary consumption over the preceding 12 months (Liu et al., 2020). Dietary exposures included nutrient-based variables (e.g., percentage of energy intake from fat, carbohydrates, and protein; total energy intake; saturated fat; dietary fiber) and selected food group variables (e.g., fruits, vegetables, grains, dairy, and meat). All dietary variables were analyzed as continuous variables. For macronutrient exposures calculated as percentages of total daily energy, no further mathematical adjustment was performed, as these metrics inherently control for total energy scale. However, to eliminate confounding by absolute caloric volume for absolute food group variables (measured in servings/day) and weight-based nutrients (g/day), a standard multivariate nutrient density model approach was implemented. Total energy intake (TEI, kcal/day) was explicitly entered as a continuous covariate in these models, thereby holding total caloric intake constant and isolating the true compositional effect of individual food categories independent of absolute energy consumption scale.

### Covariates

Covariates were selected a priori based on clinical relevance and prior literature. The primary adjusted models included age, sex, body mass index (BMI), baseline effusion-synovitis volume, and baseline KLG.

To assess the robustness of findings, additional covariates were incorporated in sensitivity analyses, including physical activity (Physical Activity Scale for the Elderly, PASE) and diabetes status. Variables considered to lie on the causal pathway, such as structural MRI features or symptom measures, were not included to avoid overadjustment bias.

### Statistical analysis

All analyses were conducted at the knee level. Because some participants contributed data from both knees, resulting in correlated observations, LMMs with participant-specific random intercepts were used to account for within-subject correlation.

For each dietary exposure, separate models were fitted:

$$\Delta {\text{ES}}_{\text{ij}}=\beta_{0}+\beta_{1}X_{\text{ij}}+\beta_{2}\text{Covariates}+u_{i}+\epsilon_{\text{ij}}$$

Where i denotes participants and j denotes knees, $u_{i}$ represents a random intercept for each participant, and $\epsilon_{\text{ij}}$ is the residual error.

Effect estimates are presented as β coefficients with 95% confidence intervals (CIs). To account for multiple testing across dietary variables, false discovery rate (FDR) correction was applied using the Benjamini–Hochberg method.

Several sensitivity analyses were conducted to evaluate the robustness of findings: (1) alternative covariate adjustment models (excluding KLG and additionally adjusting for PASE and diabetes), (2) winsorized ΔES to assess the influence of extreme values, and (3) generalized estimating equation (GEE) models with a logit link function to examine binary effusion-synovitis progression while accounting for within-subject correlation. Furthermore, to address potential confounding driven by initial variations in baseline joint inflammation levels (as recommended by the reviewer), a complementary sensitivity analysis was performed using the relative percentage change in effusion-synovitis volume as an alternative dependent variable, calculated as: $Relative\;\Delta \;ES=\left(\frac{{ES}_{12month}-{ES}_{baseline}}{{ES}_{baseline}}\right)\times 100\%$ . This relative continuous outcome was modeled using identical linear mixed-effects specifications, concurrently adjusting for age, sex, BMI, baseline ES volume, and KLG as fixed effects, with participant-specific random intercepts.

In addition to the primary longitudinal analysis, cross-sectional associations between baseline dietary factors and baseline effusion-synovitis volume were examined as complementary analyses using LMMs.

All analyses were performed using R (version 4.4.2) and Python (version 3.10). Statistical significance was defined as a two-sided p-value <0.050.

## Results

### Baseline characteristics of included knees

A total of 659 knees from 620 participants were included in the analysis. The mean age of participants was 60.4 ± 8.4 years, and 66.2% were female. The average BMI was 28.4 ± 4.4 kg/m^2^. The mean baseline effusion-synovitis volume was 5.9 ± 3.2 mL.

In terms of radiographic status, 38.1% of knees had KLG 0, while 61.9% had KLG 1. The mean physical activity level, assessed by the PASE score, was 171.6 ± 83.6, and 6.9% of participants had diabetes at baseline (Table 1).

**TABLE 1:** Baseline characteristics of included knees.

Characteristic	Overall (n = 659 knees)
Age, years	60.4 ± 8.4
Female, n (%)	429 (66.2)
BMI, kg/m²	28.4 ± 4.4
Baseline effusion-synovitis volume, mL	5.9 ± 3.2
KLG, n (%)	
Grade 0	247 (38.1)
Grade 1	401 (61.9)
Physical activity (PASE score)	171.6 ± 83.6
Diabetes, n (%)	45 (6.9)

Continuous variables are presented as mean ± standard deviation, and categorical variables as number (percentage). BMI, body mass index; KLG, Kellgren-Lawrence grade; PASE, Physical Activity Scale for the Elderly.

### Cross-sectional associations between dietary factors and baseline effusion-synovitis volume

In cross-sectional analyses, the associations between baseline dietary factors and baseline effusion-synovitis volume were evaluated using LMMs accounting for within-subject correlation (Supplementary Table S1). After adjustment for age, sex, BMI, and baseline KLG, and total energy intake (kcal/day), higher intake of meat, fish, poultry, eggs, and legumes was significantly associated with a higher baseline effusion-synovitis volume (β = 0.452, 95% CI 0.075 to 0.829, p = 0.019). However, this cross-sectional association was attenuated and did not maintain robust statistical significance after applying the conservative Benjamini–Hochberg FDR correction for multiple testing (FDR-adjusted p > 0.050) (Supplementary Table S1).

### Primary longitudinal associations between dietary factors and effusion-synovitis progression

In longitudinal analyses, the associations between baseline dietary factors and 12-month change in effusion-synovitis volume (ΔES) were evaluated using LMMs (Table 2). After adjustment for age, sex, BMI, baseline effusion-synovitis volume, and baseline KLG, none of the dietary variables demonstrated a statistically significant association with ΔES.Specifically, the percentage of energy intake from fat, which showed a nominal but non-significant inverse trend in this sample (β = −0.005, 95% CI -0.150 to 0.006, p = 0.383), did not reach statistical significance. Similarly, other dietary exposures, including food group measures (fruit, vegetables, grains, dairy, and meat), macronutrient composition (carbohydrates and protein), and nutrient-derived variables (total energy intake, dietary fiber, and saturated fat), showed no significant associations with 12-month changes in synovial inflammation (all p > 0.050). After applying the FDR correction to account for multiple testing, all associations remained non-significant (all FDR-adjusted p > 0.050) (Table 2).

**TABLE 2:** Longitudinal associations between dietary factors and 12-month change in effusion-synovitis volume (ΔES).

Variable	β (95% CI)	*P* value	FDR-adjusted P value
Percentage of energy intake from fat	-0.005 (-0.015 to 0.006)	0.383	0.986
Fruit and fruit juice intake (servings/day)	0.020 (-0.072 to 0.112)	0.668	0.986
Vegetable intake (servings/day)	-0.002 (-0.037 to 0.034)	0.931	0.986
Grain intake (servings/day)	0.001 (-0.049 to 0.050)	0.986	0.986
Dairy intake (servings/day)	0.001 (-0.008 to 0.009)	0.931	0.986
Meat, fish, poultry, eggs, and legumes intake (servings/day)	-0.036 (-0.119 to 0.047)	0.398	0.986
Total energy intake (kcal/day)	-0.0001 (-0.001 to 0.001)	0.757	0.986
Percentage of energy intake from carbohydrates	0.002 (-0.007 to 0.011)	0.645	0.986
Percentage of energy intake from protein	-0.003 (-0.026 to 0.020)	0.790	0.986
Percentage of energy intake from sweets/desserts	0.001 (-0.008 to 0.009)	0.931	0.986
Dietary fiber intake (g/day)	0.004 (-0.007 to 0.015)	0.491	0.986
Saturated fat intake (g/day)	-0.003 (-0.013 to 0.006)	0.448	0.986

Effect estimates are presented as β coefficients with their corresponding 95% CI, where the unit of β represents the absolute change in effusion-synovitis volume (in milliliters, mL) per each single unit increase in the dietary exposure (i.e., per 1 serving/day increase for food groups, per 1 g/day increase for weight-based nutrients, or per 1% increase for energy percentages). Models were adjusted for age, sex, BMI, baseline effusion-synovitis volume, and baseline KLG, with participant-specific random intercepts to account for within-subject correlation due to bilateral knees. Effect estimates are presented as β coefficients with 95% CI. FDR correction was applied using the Benjamini-Hochberg method. All dietary variables were analyzed as continuous variables. BMI, body mass index; KLG, Kellgren-Lawrence grade; CI, confidence intervals; FDR, false discovery rate.

### Robustness of the association between dietary fat intake and ΔES

The robustness of the association between dietary fat intake and ΔES was evaluated across multiple sensitivity analyses to ensure that the findings were not influenced by model specification or outcome distribution (Table 3). The lack of association between the percentage of energy intake from fat and ΔES remained consistent across all models with alternative covariate adjustments. Specifically, no significant relationship was observed when excluding KLG from the models or when additionally adjusting for physical activity (PASE score) and diabetes status (all p > 0.050). Furthermore, the results remained non-significant when extreme values of ΔES were accounted for using winsorization (e.g., β = −0.001, 95% CI -0.009 to 0.007, p = 0.816). Similarly, in analyses using a binary definition of effusion-synovitis progression (progression vs. no progression), dietary fat intake was not significantly associated with the risk of progression (e.g., odds ratio = 1.010, 95% CI 0.981 to 1.039, p = 0.516) (Table 3).

**TABLE 3:** Sensitivity analyses for the association between dietary fat intake and 12-month change in effusion-synovitis volume (ΔES).

Model	β / OR (95% CI)	*P* value
Primary model	-0.005 (-0.015 to 0.006)	0.383
Excluding KLG	-0.005 (-0.015 to 0.006)	0.383
+ Physical activity (PASE)	-0.005 (-0.015 to 0.006)	0.389
+ Diabetes	-0.006 (-0.016 to 0.005)	0.308
+ PASE + Diabetes	-0.005 (-0.016 to 0.005)	0.314
Winsorized ΔES	-0.001 (-0.009 to 0.007)	0.816
Binary progression (GEE)	1.009 (0.981 to 1.039)	0.516

Effect estimates are presented as β coefficients with their corresponding 95% CI, where the unit of βrepresents the relative percentage change in effusion-synovitis volume (%) per each single unit increase in the dietary exposure (i.e., per 1 serving/day increase for food groups, per 1 g/day increase for weight-based nutrients, or per 1% increase for energy percentages). The primary model was adjusted for age, sex, BMI, baseline effusion-synovitis volume, and baseline KLG, with participant-specific random intercepts. Alternative models included exclusion of KLG, additional adjustment for physical activity (PASE) and diabetes, winsorization of ΔES (1st-99th percentiles), and GEE models for binary progression (ΔES > 0 vs. ≤ 0). BMI, body mass index; KLG, Kellgren-Lawrence grade; GEE, generalized estimating equation; PASE, Physical Activity Scale for the Elderly.

**FIGURE 2:**
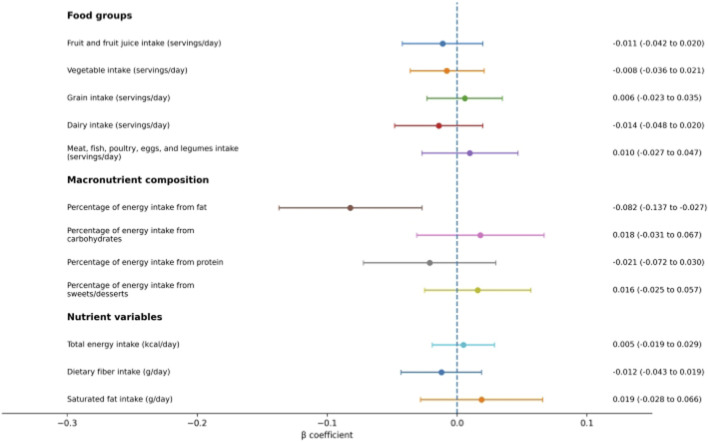
Associations between dietary factors and 12-month change in effusion-synovitis volume. Forest plot showing regression coefficients (β) and 95% CI for the associations between baseline dietary factors and 12-month change in effusion-synovitis volume (ΔES). Estimates were derived from LMMs adjusted for age, sex, BMI, baseline effusion-synovitis volume, and baseline KLG, with participant-specific random intercepts to account for within-subject correlation due to bilateral knees. The vertical dashed line indicates no association (β = 0). All dietary variables were analyzed as continuous variables. Abbreviations: CI, confidence intervals; LMMs, linear mixed-effects models; BMI, body mass index; KLG, Kellgren-Lawrence grade.

Importantly, when re-running the linear mixed-effects models using the relative percentage change in effusion-synovitis volume ( Relative Δ ES ) as the dependent variable to account for baseline volumetric variations, our core conclusions remained completely unaltered. None of the twelve evaluated dietary exposures, including macronutrient compositions, total caloric intake, or specific food groups, demonstrated a statistically significant longitudinal association with the relative percentage change in synovial inflammation (all FDR-adjusted p > 0.050; detailed estimates are provided in Figure 2 and Supplementary Table S2).

### Interaction analyses with isometric leg strength

To further align our findings with the musculoskeletal aging framework and explore potential effect modification by localized functional motor status, we performed formal interaction analyses between baseline dietary exposures and baseline isometric leg strength (variable: v00llwgt and v00rlwgt from AllClinical00. txt file in OAI database, measured in Newtons, N). Expanded linear mixed-effects models incorporating a multiplicative interaction term ( Dietary Exposure×Isometric Strength ) were fitted within the isocaloric multivariate density framework, fully adjusting for age, sex, BMI, KLG, and total energy intake. The analysis revealed no statistically significant multiplicative interaction on either baseline effusion-synovitis volume (β = 0.001, 95% CI: −0.030 to 0.033, $P_{interaction}$ = 0.927) or prospective 12-month relative percentage changes in joint fluid (β = 0.063, 95% CI: −0.225 to 0.351, $P_{interaction}$ = 0.668). These rigorous data demonstrate that the observed dietary links—and the lack of longitudinal prospective associations—are highly uniform across the entire spectrum of quadriceps muscle competence, showing no significant modification by individual physical weakness or sarcopenic tendencies. The comprehensive statistical parameters for these interaction models are compiled in Supplementary Table S3.

## Discussion

In this longitudinal knee-level analysis from the OAI, we found that dietary factors were consistently not associated with short-term changes in MRI-defined effusion-synovitis volume. Unlike some previous reports suggesting potential dietary influences on joint health, our study found no statistically significant relationship between any baseline dietary factors—including macronutrient composition and specific food groups—and 12-month effusion-synovitis progression. Notably, even the percentage of energy intake from fat, which we hypothesized might influence synovial inflammation, showed no significant association with ΔES across the primary analysis and all subsequent sensitivity analyses (including alternative covariate adjustments and outcome winsorization). Overall, our results provide robust evidence that baseline dietary intake, as measured by a food frequency questionnaire, does not meaningfully influence short-term changes in synovial inflammation in knees at risk of KOA. These findings underscore the complexity of inflammatory mechanisms in early KOA and suggest that such processes may be driven more strongly by factors other than current dietary patterns.

Previous studies have suggested that dietary patterns and nutrient intake may influence the risk and progression of KOA through systemic metabolic (Xu et al., 2021) and inflammatory pathways (Kasprzyk et al., 2025). Diets high in saturated fat and refined carbohydrates have been linked to low-grade inflammation, while diets rich in fruits, vegetables, and antioxidants have been associated with anti-inflammatory effects (Liu et al., 2020). However, most prior studies have focused on clinical outcomes such as pain, function, or incident radiographic KOA (Kasprzyk et al., 2025). In contrast, our study leveraged quantitative MRI to specifically examine effusion-synovitis volume—a more proximal, objective, and sensitive marker of local joint inflammatory activity. Our findings, which show a lack of longitudinal association between diet and 12-month inflammatory progression, contrast with some earlier studies focused on symptomatic or radiographic endpoints. This discrepancy highlights the critical importance of distinguishing between symptom-based markers and imaging-defined inflammatory phenotypes. It further suggests that while dietary factors may correlate with the clinical experience of KOA, their influence on the short-term structural fluctuations of synovial inflammation may be more limited or require a longer duration of exposure to manifest as measurable volume changes.

Several explanations may account for the lack of robust associations observed in this study. First, synovial inflammation in KOA is a complex and multifactorial process influenced by mechanical loading, structural alterations, and systemic metabolic factors (Sanchez-Lopez et al., 2022). Crucially, this discrepancy may be rooted in a chronological phenomenon of “temporal decoupling” between the retrospective dietary exposure assessment and the subsequent window of disease progression. Because the baseline Block Brief 2000 FFQ captures habitual dietary habits over the preceding 12 months, this long-term exposure has undergone a sufficient behavioral “incubation period” to actively shape the existing metabolic and chronic inflammatory “set-point” of the knee joint at baseline. This perfectly explains why dietary variables, such as meat and legume intake, correlate significantly with baseline effusion-synovitis volume. In contrast, evaluating 12-month prospective shifts ($\Delta$ES) relies on a static baseline exposure metric to predict a highly dynamic imaging biomarker. Effusion-synovitis reflects transient, fluctuating inflammatory states that are sensitive to acute biomechanical loading shifts or proximal lifestyle changes occurring during the follow-up year. Without sequential, time-varying dietary tracking between baseline and the 12-month visit, potential temporal variations in participant diet remain unmeasured, decoupling the baseline exposure data from the active fluid fluctuations. Dietary intake alone may have a relatively modest effect on local joint inflammation, particularly over a short follow-up period (Neogi, 2013). Second, effusion-synovitis is a dynamic imaging biomarker that reflects transient inflammatory changes which can fluctuate significantly over time. Such temporal variability may limit the power of a single baseline dietary assessment to predict long-term trajectories of synovial volume change. Third, although the semi-automated MRI segmentation used in this study provides high precision, the inherent measurement variability in both the Block Brief FFQ (which relies on participant recall) and MRI-derived volumetric assessments may have attenuated the observed longitudinal effect sizes. Finally, the exclusion of participants who developed incident radiographic KOA within the first year—while necessary to isolate inflammatory changes from structural progression—may have resulted in a study sample with more stable joint environments, further reducing the likelihood of detecting diet-induced inflammatory shifts in the short term.

To ground our analysis within the musculoskeletal aging framework, we evaluated potential effect modification by localized functional status using baseline isometric leg strength—a validated surrogate for quadriceps competence and a clinical hallmark of sarcopenia. Multiplicative interaction analyses within our isocaloric framework revealed no statistically significant interaction on either baseline effusion-synovitis volume (β = 0.001, $P_{interaction}$ = 0.927) or 12-month prospective relative fluid changes (β = 0.063, $P_{interaction}$ = 0.668). In the paradigm of sarcopenic obesity, synchronized muscle loss and adiposity typically accelerate joint degradation via systemic adipokine toxicity and localized mechanical misalignment. The absence of a significant interaction here suggests a mechanical-metabolic decoupling at this early, pre-radiographic stage of joint inflammation. While dietary components independently influence the steady-state baseline metabolic ‘set-point’ of the synovium, prospective short-term volumetric shifts (ΔES) appear dominantly governed by immediate biomechanical constraints and quadriceps-driven stability rather than active muscle-diet synergistic cross-talk. This independent segregation highlights that preserving quadriceps strength remains an irreplaceable biomechanical countermeasure for aging populations, functioning independently of nutritional variations.

Importantly, while we observed a cross-sectional association between specific protein sources and baseline synovial volume, the overall absence of robust associations in longitudinal analyses suggests that dietary factors may not be strongly linked to the short-term progression of synovial inflammation (Shawl et al., 2024; Sanchez-Lopez et al., 2022). This discrepancy between cross-sectional and longitudinal findings aligns with observations in other imaging-based KOA studies, where dietary influences often appear more pronounced in baseline disease states than in rapid structural change (Wang et al., 2016a; Wang et al., 2016b; Wang et al., 2016c). Such consistency across our various longitudinal sensitivity models strengthens the interpretation that dietary patterns, as captured at baseline, have a limited impact on 12-month inflammatory trajectories.

The nominal inverse association between fat-derived energy intake and ΔES initially observed in our primary analysis should be interpreted with extreme caution (Sophocleous, 2023). As demonstrated in our sensitivity analyses, this finding was highly sensitive to the influence of extreme values (outliers) and failed to reach significance in winsorized or categorical progression models (Sophocleous, 2023; Carnoli et al., 2024). This underscores the critical importance of comprehensive sensitivity analyses in nutritional epidemiology, as extreme dietary reporting or unusual inflammatory fluctuations can easily produce spurious associations that lack clinical stability (Carnoli et al., 2024).

From a clinical perspective, our findings suggest that dietary modification alone may have a limited impact on the short-term mitigation of MRI-defined synovial inflammation in individuals at risk of KOA (Kasprzyk et al., 2025; Bruder et al., 2026). This does not preclude a role for diet in broader disease pathways—such as pain modulation or systemic metabolic health—but rather highlights that local inflammation detectable by MRI may be driven more dominantly by biomechanical loading and joint structural integrity rather than immediate nutrient intake (Sanchez-Lopez et al., 2022). Notably, the significant link between meat and legume intake and baseline ES volume warrants further investigation, as high-protein diets have been previously associated with altered metabolic profiles and systemic inflammatory markers like C-reactive protein (CRP) in aging populations (Kasprzyk et al., 2025; Kloppenburg et al., 2025). Future studies should prioritize longer-term longitudinal assessments, the role of specific dietary patterns (e.g., the Mediterranean diet), and the complex interactions between nutrition, the gut microbiome, and biomechanical stress (Sanchez-Lopez et al., 2022).

Several limitations of this study warrant acknowledgment. First, dietary intake was assessed using the FFQ at a single time point. Although validated, FFQs are inherently subject to recall bias and measurement error, which may lead to the misclassification of dietary exposures and potentially attenuate observed longitudinal associations. Importantly, because this questionnaire reflects retrospective diet over the preceding 12 months, the lack of time-varying dietary assessments during follow-up introduces a chronological “temporal decoupling.” This timeline mismatch means that while retrospective habits had an adequate behavioral “incubation period” to manifest as the baseline inflammatory “set-point,” concurrent dietary shifts during the follow-up year remained uncaptured, limiting the capacity to predict active prospective fluid trajectories. Second, the 12-month follow-up period may be insufficient to detect the cumulative, longer-term effects of dietary patterns on structural changes in synovial tissue, which often evolve over several years. Third, despite adjusting for key covariates like BMI and KLG, residual confounding from unmeasured factors—such as detailed systemic metabolic profiles, gut microbiota composition, or specific inflammatory biomarkers like CRP—cannot be entirely excluded. Such factors could potentially mediate or confound the cross-sectional relationship observed between protein-rich foods and baseline inflammation. Finally, as this was a secondary analysis of the imaging-enriched POMA sub-cohort, the findings may have limited generalizability to the broader, general population or to individuals with established advanced radiographic KOA. These limitations notwithstanding, the use of quantitative MRI and rigorous sensitivity analyses provides a high level of internal validity for the inflammatory phenotypes assessed in this study.

## Conclusion

In conclusion, this longitudinal MRI-based study demonstrates that while certain dietary factors, specifically higher intake of meat and legumes, are associated with greater baseline effusion-synovitis volume, there is no robust evidence that baseline dietary intake is associated with the short-term progression of synovial inflammation over 12 months in knees at risk of KOA.

## Data Availability

The datasets presented in this study can be found in online repositories. The names of the repository/repositories and accession number(s) can be found in the article/Supplementary Material.
