# Supplementary material for: Dietary factors and MRI-Defined synovial inflammation progression in knee osteoarthritis: a longitudinal mixed-effects analysis from the osteoarthritis initiative
Source: Front Aging. 2026 Jul 29;7:1877396. doi: 10.3389/fragi.2026.1877396 (PMC13461594; doi:10.3389/fragi.2026.1877396)
Supplement: Supplementary file 1 [file Supplementaryfile2.docx]

**Supplementary materials**

Supplementary Figure S1

Supplementary Table S1

Supplementary Table S2

Supplementary Table S3

Figure S1

**Table S1. Cross-sectional associations between dietary factors and baseline effusion-synovitis volume.**

| **Variable** | **β (95% CI)** | **P value** | **FDR-adjusted P** |
| --- | --- | --- | --- |
| **Macronutrient Percentages (Energy-Self-Adjusting)** |  |  |  |
| Percentage of energy intake from fat | 0.006 (-0.028 to 0.040) | 0.733 | >0.050 |
| Fruit and fruit juice intake (servings/day) | -0.025 (-0.321 to 0.271) | 0.869 | >0.050 |
| Vegetable intake (servings/day) | 0.092 (-0.022 to 0.206) | 0.112 | >0.050 |
| Grain intake (servings/day) | 0.133 (-0.026 to 0.291) | 0.100 | >0.050 |
| Dairy intake (servings/day) | -0.009 (-0.036 to 0.019) | 0.529 | >0.050 |
| Meat, fish, poultry, eggs, and legumes intake (servings/day) | 0.443 (0.178 to 0.707) | 0.001 | 0.012 |
| Total energy intake (kcal/day) | 0.001 (0.0001 to 0.001) | 0.023 | >0.050 |
| Percentage of energy intake from carbohydrates | -0.013 (-0.041 to 0.015) | 0.372 | >0.050 |
| Percentage of energy intake from protein | 0.017 (-0.057 to 0.091) | 0.660 | >0.050 |
| Percentage of energy intake from sweets/desserts | -0.009 (-0.036 to 0.019) | 0.529 | >0.050 |
| Dietary fiber intake (g/day) | 0.043 (0.007 to 0.079) | 0.019 | >0.050 |
| Saturated fat intake (g/day) | 0.028 (-0.001 to 0.057) | 0.060 | >0.050 |
| **Absolute Food Groups & Nutrients (Isocalorically Adjusted for TEI)** |  |  |  |
| Meat, fish, poultry, eggs, and legumes intake (servings/day) | 0.452 (0.075 to 0.828) | 0.019 | >0.050 |
| Fruit and fruit juice intake (servings/day) | -0.170 (-0.486 to 0.146) | 0.292 | >0.050 |
| Vegetable intake (servings/day) | 0.044 (-0.082 to 0.170) | 0.492 | >0.050 |
| Grain intake (servings/day) | 0.031 (-0.171 to 0.232) | 0.765 | >0.050 |
| Dairy intake (servings/day) | -0.016 (-0.044 to 0.012) | 0.267 | >0.050 |
| Dietary fiber intake (g/day) | 0.027 (-0.022 to 0.076) | 0.281 | >0.050 |
| Saturated fat intake (g/day) | -0.004 (-0.061 to 0.052) | 0.887 | >0.050 |
| Total energy intake (kcal/day) | 0.001 (0.00002 to 0.001) | 0.023 | >0.050 |

Estimates were derived from linear mixed-effects models (LMMs) with participant-specific random intercepts to account for within-subject correlation due to bilateral knees. All models were adjusted for baseline age, sex, BMI, and KLG. Effect estimates are presented as β coefficients with their corresponding 95% CI, where the unit of β represents the absolute change in effusion-synovitis volume (in milliliters, mL) per each single unit increase in the dietary exposure (i.e., per 1 serving/day increase for food groups, per 1 g/day increase for weight-based nutrients, or per 1% increase for energy percentages). Models evaluating absolute food groups (servings/day) and nutrient weights (g/day) were additionally adjusted for absolute total energy intake (TEI, kcal/day) as a continuous covariate (the multivariate nutrient density approach) to maintain an isocaloric framework. Total energy intake was evaluated in a model containing standard non-dietary covariates only. FDR correction was applied across all 12 primary dietary exposures using the Benjamini-Hochberg method. Effect estimates are presented as β coefficients with 95% CI. FDR correction was applied using the Benjamini-Hochberg method. BMI, body mass index; KLG, Kellgren-Lawrence grade; CI, confidence intervals; FDR, false discovery rate; TEI, total energy intake.

Table S2. Sensitivity analysis evaluating longitudinal associations between baseline dietary factors and 12-month relative percentage change in effusion-synovitis volume (Relative ∆ ES).

| **Variable** | **β Coefficient (95% Confidence Interval)** | **Unadjusted P value** | **FDR-adjusted P** |
| --- | --- | --- | --- |
| **Macronutrients & Energy Intake** |  |  |  |
| Percentage of energy intake from fat | -0.040 (-0.333 to 0.254) | 0.792 | >0.050 |
| Percentage of energy from carbohydrates | 0.083 (-0.157 to 0.323) | 0.499 | >0.050 |
| Percentage of energy from protein | 0.0003 (-0.638 to 0.639) | 0.999 | >0.050 |
| Total energy intake (kcal/day) | 0.001 (-0.004 to 0.005) | 0.781 | >0.050 |
| Saturated fat intake (g/day) | -0.0004 (-0.251 to 0.250) | 0.997 | >0.050 |
| **Food Groups & Specific Components** |  |  |  |
| Fruit and fruit juice intake (servings/day) | 0.880 (-1.678 to 3.439) | 0.502 | >0.050 |
| Vegetable intake (servings/day) | 0.210 (-0.783 to 1.204) | 0.678 | >0.050 |
| Grain intake (servings/day) | 0.398 (-0.976 to 1.771) | 0.571 | >0.050 |
| Dairy intake (servings/day) | -0.034 (-0.271 to 0.204) | 0.781 | >0.050 |
| Meat, fish, poultry, eggs, legumes (servings/day) | -0.388 (-2.712 to 1.936) | 0.743 | >0.050 |
| Percentage of energy from sweets/desserts | -0.034 (-0.271 to 0.204) | 0.781 | >0.050 |
| Dietary fiber intake (g/day) | 0.140 (-0.173 to 0.454) | 0.381 | >0.050 |

Estimates were derived from separate linear mixed-effects models (LMMs) with participant-specific random intercepts to account for within-subject correlation due to bilateral knees. Effect estimates are presented as β coefficients with their corresponding 95% CI, where the unit of βrepresents the relative percentage change in effusion-synovitis volume (%) per each single unit increase in the dietary exposure (i.e., per 1 serving/day increase for food groups, per 1 g/day increase for weight-based nutrients, or per 1% increase for energy percentages). All models were simultaneously adjusted for baseline covariates including age, sex, body mass index (BMI), baseline effusion-synovitis volume, and baseline Kellgren-Lawrence grade (KLG). The dependent variable is defined as: $elative \Delta ES = \left( \frac{{ES}_{12month}-{ES}_{baseline}}{{ES}_{baseline}} \right)\times100\%$. Multiple testing correction was performed using the Benjamini-Hochberg false discovery rate (FDR) method. All dietary exposures were analyzed as continuous variables. Dairy intake was calculated utilizing corresponding proxy tracking from the standardized baseline questionnaire components as detailed in the primary methods description.

Table S3. Interaction analyses between baseline dietary exposures and isometric leg strength on cross-sectional and longitudinal effusion-synovitis outcomes.

| **Analytical Model & Independent Variables** | **β Coefficient (95% CI)** | **Standard Error (SE)** | **Z-score** | **P value** |
| --- | --- | --- | --- | --- |
| Model 1: Cross-Sectional Outcome |  |  |  |  |
| (Dependent Variable: Baseline ES Volume, mL) |  |  |  |  |
| Meat, fish, poultry, eggs, and legumes intake | 0.431 (-0.214 to 1.076) | 0.329 | 1.309 | 0.191 |
| Isometric leg strength (N) | -0.022 (-0.091 to 0.047) | 0.035 | -0.617 | 0.537 |
| Meat/Legumes intake $\times$ Isometric leg strength | 0.001 (-0.030 to 0.033) | 0.016 | 0.091 | 0.927 |
|  |  |  |  |  |
| Model 2: Longitudinal Outcome |  |  |  |  |
| (Dependent Variable: 12-month Relative ∆ES, %) |  |  |  |  |
| Meat, fish, poultry, eggs, and legumes intake | -3.167 (-8.990 to 2.655) | 2.971 | -1.066 | 0.286 |
| Isometric leg strength (N) | -0.276 (-0.896 to 0.344) | 0.316 | -0.872 | 0.383 |
| Meat/Legumes intake $\times$ Isometric leg strength | 0.063 (-0.225 to 0.351) | 0.147 | 0.429 | 0.668 |

Data were analyzed at the knee level using linear mixed-effects models (LMMs) with participant-specific random intercepts to account for within-subject correlation due to bilateral knees. For Model 1 (cross-sectional), the unit of β represents the absolute change in effusion-synovitis volume (in milliliters, mL) per each single unit increase in the dietary exposure. For Model 2 (longitudinal), the unit of β represents the relative percentage change (%) in effusion-synovitis volume per each single unit increase in the dietary exposure (i.e., per 1 serving/day increase for food groups, per 1 g/day increase for weight-based nutrients, or per 1% increase for energy percentages). Model 1 (Cross-sectional) evaluated baseline effusion-synovitis volume as the dependent outcome, simultaneously adjusting for non-dietary covariates including age, sex, body mass index (BMI), and KLG, alongside total energy intake (kcal/day) to maintain an isocaloric multivariate density framework. Model 2 (Longitudinal) evaluated the 12-month relative percentage change in effusion-synovitis volume (defined as: $Relative \Delta ES = \left( \frac{\mathrm{ES}_{12month}-\mathrm{ES}_{\mathrm{baseline}}}{\mathrm{ES}_{\mathrm{baseline}}} \right)\times100\%$) as the dependent continuous outcome, concurrently adjusting for baseline age, sex, BMI, KLG, baseline effusion-synovitis volume, and total energy intake (TEI, kcal/day). CI, confidence interval; ES, effusion-synovitis; KLG, Kellgren-Lawrence grade.
